# Characterization of the equine skeletal muscle transcriptome identifies novel functional responses to exercise training

**DOI:** 10.1186/1471-2164-11-398

**Published:** 2010-06-23

**Authors:** Beatrice A McGivney, Paul A McGettigan, John A Browne, Alexander CO Evans, Rita G Fonseca, Brendan J Loftus, Amanda Lohan, David E MacHugh, Barbara A Murphy, Lisa M Katz, Emmeline W Hill

**Affiliations:** 1 Animal Genomics Laboratory, UCD School of Agriculture, Food Science and Veterinary Medicine, College of Life Sciences, University College Dublin, Belfield, Dublin 4, Ireland; 2 University Veterinary Hospital, UCD School of Agriculture, Food Science and Veterinary Medicine, College of Life Sciences, University College Dublin, Belfield, Dublin 4, Ireland; 3 UCD Conway Institute of Biomolecular and Biomedical Research, University College Dublin, Belfield, Dublin 4, Ireland

## Abstract

**Background:**

Digital gene expression profiling was used to characterize the assembly of genes expressed in equine skeletal muscle and to identify the subset of genes that were differentially expressed following a ten-month period of exercise training. The study cohort comprised seven Thoroughbred racehorses from a single training yard. Skeletal muscle biopsies were collected at rest from the *gluteus medius *at two time points: T_1 _- untrained, (9 ± 0.5 months old) and T_2 _- trained (20 ± 0.7 months old).

**Results:**

The most abundant mRNA transcripts in the muscle transcriptome were those involved in muscle contraction, aerobic respiration and mitochondrial function. A previously unreported over-representation of genes related to RNA processing, the stress response and proteolysis was observed. Following training 92 tags were differentially expressed of which 74 were annotated. Sixteen genes showed increased expression, including the mitochondrial genes *ACADVL*, *MRPS21 *and *SLC25A29 *encoded by the nuclear genome. Among the 58 genes with decreased expression, *MSTN*, a negative regulator of muscle growth, had the greatest decrease.

Functional analysis of all expressed genes using FatiScan revealed an asymmetric distribution of 482 Gene Ontology (GO) groups and 18 KEGG pathways. Functional groups displaying highly significant (*P *< 0.0001) increased expression included mitochondrion, oxidative phosphorylation and fatty acid metabolism while functional groups with decreased expression were mainly associated with structural genes and included the sarcoplasm, laminin complex and cytoskeleton.

**Conclusion:**

Exercise training in Thoroughbred racehorses results in coordinate changes in the gene expression of functional groups of genes related to metabolism, oxidative phosphorylation and muscle structure.

## Background

The phenotypic and biochemical changes occurring in response to exercise training have been extensively studied in humans and mammals, the results mainly being of a descriptive nature. The adaptive response to training is dependent on variations in exercise-induced changes in muscle load, energy requirements and calcium flux. Endurance training results in increased aerobic capacity [1], mitochondrial biogenesis [2] and a shift from carbohydrate to fat metabolism [3] whereas resistance training promotes protein synthesis [4,5], muscle hypertrophy [6] and a switch from slow to fast twitch muscle. Numerous equine studies have also confirmed an increase in VO_2max _and an increase in oxidative enzyme activity [7-12] following endurance training. An increase in type II and a concurrent decrease in type IIX fibres is observed in Thoroughbreds in response to high intensity training [13,14]. Also, anerobic capacity and speed and strength have been observed to increase following short duration high intensity (~100-150% VO_2max_) exercise [14-16].

In contrast, much less is known regarding the transcriptional reprogramming underlying the highly specific adaptive responses to endurance and resistance exercise. Exercise studies using human subjects and animal models have demonstrated that changes in the expression of a wide range of mRNA transcripts play a major role in the recovery of muscle following exercise with the expression levels of most genes returning to baseline within 24 hours [17-23]. However, it appears that repeated bouts of exercise lead to new basal levels of gene expression in resting muscle. Higher levels of mitochondrial genes and genes involved in energy metabolism were observed in endurance trained athletes compared to sedentary subjects [24]. Further evidence for a new steady state level of exercise related genes comes from a recent study in which differential levels of gene expression were observed in resting skeletal muscle from sedentary, endurance trained and resistance trained subjects. However the use of intra rather than inter-individual genetic comparisons as well as different training stimuli may have contributed to the observed differences in gene expression between the groups. The majority of differentially expressed genes were common to both trained states [25]. A surprisingly small number of genes were differentially expressed between endurance trained and resistance trained subjects given the very different phenotypic changes and distinct signalling pathways [26,27] associated with each form of exercise. Studies have indicated that concurrent endurance and resistance training results in impaired strength development and aerobic capacity when compared to a training regime with a single exercise mode [28-31] a phenomenon described as the interference effect. However, conflicting studies have found little or no effect of a combined training regime on strength and aerobic capacity [32-35]. The aim of this study was to investigate the global transcriptional response in skeletal muscle to a training regime combining endurance and high intensity sprint exercise in Thoroughbred racehorses. We hypothesise that following training differential expression of genes related to both aerobic capacity and muscle hypertrophy will be observed reflecting the dual nature of the training regime.

The Thoroughbred is a novel and valuable model for identifying molecular mechanisms basic to both endurance and resistance adaptive responses. Competitive horse racing dates to 4500 BC and Thoroughbreds have been bred for speed and stamina since the 1700 s. This intense selection has resulted in a highly adapted athlete [36]. Thoroughbreds have a very high aerobic capacity or maximal oxygen uptake (VO_2max_) [37] relative to their body mass. For instance, VO_2max _can reach 180-200 mL O_2_/min/kg, approximately 2.5 times higher than other species of similar size[38]. This is achieved through a large lung volume, high cardiac output, high haemoglobin concentration, high muscle mitochondrial volume and a high skeletal muscle mass [38-44]. During intense exercise such as under racing conditions a Thoroughbred may increase its metabolic rate from basal levels by up to 60-fold [45]. Furthermore, the Thoroughbred has a very high skeletal muscle mass comprising over 55% of total body mass [46].

A Thoroughbred racehorse trained for flat racing undergoes a training regime comprising intermittent days of sprint exercise to promote increased muscle mass among periods of prolonged exercise at a slower pace to enhance aerobic capacity. In a previous study we detected molecular signatures of both endurance and resistance exercise in untrained Thoroughbred skeletal muscle following a single bout of exhaustive exercise [47]. A further advantage of using Thoroughbreds as an exercise model is that inter-individual comparisons can be made between subjects that come from a similar background (genetic and environmental) and have undergone a similar exercise regime with relatively little variation in management. Variations in genetic and environmental conditions cannot be controlled to the same extent in human subjects.

To-date the main approach to investigating global transcriptional changes has been the use of gene expression microarray platforms [47-49]. In this study we have used digital gene expression (DGE, Illumina) profiling to characterize the assembly of genes expressed in equine skeletal muscle and to identify the subset of genes that were differentially expressed following a ten month period of exercise training. DGE is a recently developed alternative to microarray gene expression profiling [50-52]. The DGE method involves the generation of a cDNA library with a 17 bp tag generated by restriction digestions for each mRNA transcript. The tags are directly sequenced using the Illumina Genome Analyzer creating millions of short reads. In contrast to microarray technology which is limited to the hybridisation of cDNA to probes printed on the array platform, DGE is not dependent on currently available genome sequence and thus provides a global, hypothesis-free quantitative analysis of the transcriptome. The technique is conceptually similar to serial analysis of gene expression (SAGE) [53] but substantially less expensive, more general and capable of delivering more information.

Using this technique we investigated 1) the overrepresentation of functional groups in skeletal muscle relative to the entire genome, 2) the genes differentially expressed in trained relative to untrained skeletal muscle, and 3) the overrepresentation of functional groups in genes differentially expressed following training in skeletal muscle.

## Results and discussion

### Representation of genes by DGE tags

A limitation of genome wide gene expression analysis using DGE is that it is not possible to evaluate the expression of genes that do not contain a *NlaIII *restriction site and in some cases there is ambiguity regarding the tag-gene matches as a single tag may match to two or more genes. 91% (*n *= 22,996) of equine genes with Ensembl gene IDs (*n *= 25,180) have a *NlaIII *restriction site but 13% of these are not unique; therefore, 78% (*n *= 19,271) of currently annotated equine genes are quantifiable using DGE.

As poor quality sequence was obtained for one of the samples just 13 samples were used for analysis. Of the 13 samples successfully sequenced a total of 183 million raw reads were generated. Of these 119 million reads passed the Illumina pipeline quality filters. These 119 million usable reads consisted of 17.6 million unique tags. 66% of the usable reads mapped to the horse genome, 30% of the usable reads mapped to the predicted Ensembl gene restriction sites and 36% to the genomic regions.

The intragenic reads may represent regulatory non-coding RNAs or novel genes. However, more likely is the explanation that these tags are a combination of segments of genes that were excluded from the current annotation (or assembly) of the equine genome; an observation which has been previously reported [54] as well as tags containing sequencing errors. We expect that as the annotation of the horse transcriptome improves that most of the genomic tags we have sequenced will be reassigned to genic tags. In particular we believe that a *de novo *transcriptome assembly approach (using longer sequencing reads) of the equine muscle transcriptome would enable us to more accurately allocate tags to the correct gene models. In the absence of an accurate muscle transcriptome we believe that the Ensembl horse transcriptome, which is predominantly automatically generated and infers much of the information about gene models by homology from better annotated organisms, represents the best available option for DGE tag mapping.

The reasons that reads may not match a genomic location include ambiguous reads (same sequence tag present in more than one genomic location), reads overlapping an unannotated exon boundary, sequencing errors or single nucleotide variants present in the tag. Due to the short nature of the reads used in DGE compared to other sequencing protocols it is problematic to correct for SNPs or sequencing errors by allowing mismatched bases. Other protocols (*e.g*. RNAseq), which generate longer tags, can overcome this limitation but they introduce new problems, the most important of which is multiple tags per transcript and a bias towards highly expressed long transcripts [55]. The intragenic reads may represent regulatory non-coding RNAs or novel genes. However, more likely is the explanation that these tags represent segments of genes that were excluded from the current annotation (or assembly) of the equine genome; an observation which has been previously reported [54].

**Figure 1:**
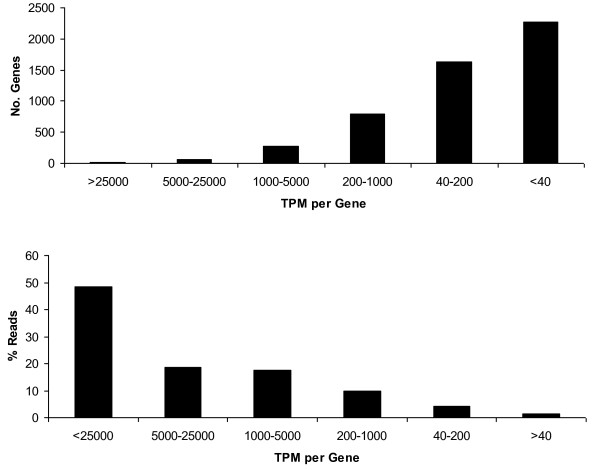
**Relationship between the number of genes expressed and mRNA abundance**. a - Number of genes compared to tags per million per gene. b - The % of the total number of reads compared to the tags per million per gene.

Only the 20% of reads that unambiguously matched Ensembl genes were used for further analysis. These represented 5,068 unique genes, ~25% of annotated equine genes. As some genes were represented by multiple different transcripts these were summed to calculate the total number of reads per gene. Highly expressed genes where > 50,000 tags per million (TPM) were detected made up 39% of all annotated reads. However, the majority of unique genes were expressed at low levels (*i.e*. 2,200 genes, < 40 TPM) and there was an inverse relationship between the level of gene expression and the number of genes expressed (Figure 1).

### Functional annotation of muscle transcriptome

Using the online tool DAVID, 448 gene ontology groups and 14 KEGG pathways were observed to be significantly (FDR = 0.05) overrepresented in skeletal muscle relative to the entire genome. There was a substantial overlap of genes within these functional groups resulting in the overrepresentation of a large number of functionally similar gene ontology groups. Therefore only the highly significant groups are shown in Table 1. The overrepresentation of mitochondrial genes, and genes involved in muscle contraction and metabolism concurs with current SAGE data [56]. However, an overrepresentation of genes related to RNA processing, the stress response and proteolysis has not, to our knowledge, previously been reported in the muscle transcriptome. DGE is much more sensitive to the detection of low level transcripts than SAGE and consequently provides greater coverage of the muscle transcriptome. When functional analysis of only the highly expressed genes (those comprising > 0.05% of annotated muscle transcriptome) was performed, the novel overrepresented functional groups were not identified. This indicates that these are comprised of genes expressed at relatively low levels. Furthermore, functional groups involved in muscle contraction and aerobic respiration were more significantly overrepresented among the highly expressed genes (Table 1).

**Table 1:** Functional groups of genes identified in the equine skeletal muscle transcriptome.

		All genes			High abundance genes		
**GO ID**	**Term**	**Count**	**FC**	**Adj P**	**Count**	**FC**	**Adj P**

GO BP:0008152	metabolic process	2468	1.19	8.17 × 10^-55^	131	1.2	4.13 × 10^-08^
GO MF:0005515	protein binding	2122	1.35	4.53 × 10^-90^	105	1.3	2.88 × 10^-12^
GO CC:0043234	protein complex	713	1.57	2.75 × 10^-43^	68	2.9	1.32 × 10^-15^
GO CC:0005739	mitochondrion	407	1.97	5.63 × 10^-49^	53	4.9	3.71 × 10^-23^
GO BP:0006936	*muscle contraction*	73	1.94	1.78 × 10^-07^	33	8.7	3.98 × 10^-17^
hsa00190	*Oxidative phosphorylation*	56	1.55	0.006	32	9.5	4.47 × 10^-18^
GO MF:0003723	RNA binding	289	1.76	3.84 × 10^-23^	27	3.3	3.78 × 10^-06^
GO MF:0009055	*electron carrier activity*	94	1.81	1.47 × 10^-07^	24	9.4	1.43 × 10^-11^
GO CC:0043292	*contractile fibre*	50	3.37	3.19 × 10^-17^	23	29.9	5.32 × 10^-18^
GO CC:0005840	*ribosome*	93	1.32	1.63 × 10^-02^	21	5.8	9.29 × 10^-09^
GO MF:0005524	ATP binding	434	1.28	1.71 × 10^-07^	20	1.2	NS
GO BP:0006950	response to stress	337	1.39	9.17 × 10^-10^	19	1.5	NS
GO CC:0044428	nuclear part	429	1.88	9.51 × 10^-46^	10	0.8	NS
GO BP:0006457	protein folding	132	2.12	5.64 × 10^-18^	10	3.1	0.007
GO BP:0015031	protein transport	290	1.85	5.91 × 10^-28^	7	0.9	NS
GO MF:0045182	translation regulator activity	76	2.40	9.13 × 10^-13^	5	3.2	NS
GO BP:0006396	RNA processing	195	1.90	8.21 × 10^-20^	4	0.8	NS
GO MF:0008134	transcription factor binding	153	1.67	1.89 × 10^-09^	4	0.9	NS
hsa00620	Pyruvate metabolism	27	2.28	3.30 × 10^-04^	4	3.6	NS
hsa00020	Citrate cycle (TCA cycle)	20	2.36	0.003	4	5.1	NS
hsa04120	Ubiquitin mediated proteolysis	55	1.49	0.016	2	0.6	NS

hsa00071	Fatty acid metabolism	24	1.89	0.017	2	1.7	NS

GO CC:0005681	spliceosome	78	2.71	2.04 × 10^-18^	1	0.7	NS
hsa03050	Proteasome	21	3.38	2.28 × 10^-07^	1	1.7	NS

GO categories and KEGG pathways overrepresented among genes expressed in equine skeletal muscle compared to all annotated equine genes. Terms in italics are more significantly overrepresented among the high abundance genes. High abundance genes are defined as those comprising > 0.05% of annotated muscle transcriptome. FC - fold change; Adj P - adjusted *P *value using the Benjamini and Hochberg method.

A list of the most abundant genes (those comprising >0.5% of annotated muscle transcriptome) is presented in Table 2. Just 28 genes contribute to over 50% of the annotated mRNA in equine skeletal muscle and are principally involved in muscle contraction and energy metabolism. Creatine kinase muscle (*CKM*) was the most abundantly expressed gene in equine skeletal muscle representing 6.9% of the annotated transcriptome and creatine kinase, mitochondrial 2, (*CKMT2*), was ranked 20^th ^among the most abundantly expressed genes, making up 0.8% of the transcriptome. Human studies using SAGE have indicated that *CKM *mRNA makes up ~1% of the human skeletal muscle transcriptome and *CKMT2 *did not feature in a list of the 54 most abundantly expressed genes [56]. The very high levels of both isoforms of creatine kinase in equine muscle compared to humans is indicative of the highly adapted athletic capacity of Thoroughbred horses as creatine kinases play a crucial role as an energy store in tissues with fluctuating energy demands. CKM is utilised during anaerobic respiration while CKMT2 is tightly coupled to oxidative phosphorylation [57-60]. The importance of CKM in athletic adaptation in the horse is further supported by the identification of a novel SNP in the *CKM *gene that, in a preliminary study, has been observed to be associated with racing performance [61]. The third most highly expressed gene in equine skeletal muscle, actin, alpha 1, skeletal muscle (*ACTA1*) has also been implicated as a candidate athletic performance gene following a genome scan for positive selection in Thoroughbred horses [62].

**Table 2:** Highly abundant transcripts in equine skeletal muscle

Gene symbol	Gene name	Total reads	% All	Cumulative %	Average no. reads pre-training	% All	Average no. reads post training	% All
*CKM*	Creatine kinase, muscle	236,942	6.9	6.9	18,642	6.7	17,870	7.1
*MYL1*	Myosin, light polypeptide 1, alkali; skeletal, fast	203,634	5.9	12.8	17,454	6.2	14,130	5.6
*ACTA1*	Actin, alpha 1, skeletal muscle	147,884	4.3	17.1	15,065	5.4	8,214	3.3
*TNNC2*	Troponin C type 2 (fast)	147,779	4.3	21.4	12,128	4.3	10,716	4.2
*ALDOA*	Aldolase A, fructose-bisphosphate	118,636	3.4	24.8	9,506	3.4	8,800	3.5
*TTN*	Titin	116,385	3.4	28.2	9,919	3.5	8,125	3.2
*MYLPF*	Myosin light chain, phosphorylatable, fast skeletal muscle	97,094	2.8	31.0	7,426	2.7	7,506	3.0
*MYH1*	Myosin, heavy polypeptide 1, skeletal muscle, adult	91,271	2.7	33.7	8,611	3.1	5,658	2.2
*TPM2*	Tropomyosin 2 (beta)	73,395	2.1	35.8	6,240	2.2	5,136	2.0
*ATP2A1*	ATPase, Ca++ transporting, cardiac muscle, fast twitch 1	59,889	1.7	37.6	5,516	2.0	3,827	1.5
*RPLP1P4*	Ribosomal protein, large, P1 pseudogene 4	55,290	1.6	39.2	4,340	1.6	4,178	1.7
*ENO3*	Enolase 3 (beta, muscle)	40,827	1.2	40.4	3,143	1.1	3,138	1.2
*LOC440359*	Similar to cold shock domain protein A short isoform	36,681	1.1	41.4	2,877	1.0	2,774	1.1
*ATP5O*	ATP synthase, H+ transporting, mitochondrial f1 complex, o subunit	34,234	1.0	42.4	2,212	0.8	2,994	1.2
*MYOZ1*	MYOZENIN 1	32,638	0.9	43.4	2,449	0.9	2,564	1.0
*PYGM*	Phosphorylase, glycogen; muscle	31,710	0.9	44.3	2,895	1.0	2,048	0.8
*TPM1*	Tropomyosin 1 (alpha)	30,823	0.9	45.2	2,820	1.0	1,986	0.8
*MYBPC2*	Myosin binding protein C, fast type	28,386	0.8	46.0	2,409	0.9	1,990	0.8
*TNNT3*	Troponin t type 3 (skeletal, fast)	27,469	0.8	46.8	1,960	0.7	2,244	0.9
*CKMT2*	Creatine kinase, mitochondrial 2 (sarcomeric)	27,394	0.8	47.6	1,622	0.6	2,523	1.0
*TNNI2*	Troponin I type 2 (skeletal, fast)	25,005	0.7	48.3	1,674	0.6	2,138	0.8
*PGK1*	Phosphoglycerate kinase 1	24,378	0.7	49.0	2,005	0.7	1,764	0.7
*RPL30*	Ribosomal protein L30	23,575	0.7	49.7	2,162	0.8	1,515	0.6
*NDUFAB1*	NADH dehydrogenase (ubiquinone) 1, alpha/beta subcomplex, 1, 8 kDa	22,285	0.6	50.4	1,570	0.6	1,838	0.7
*SLC25A4*	Solute carrier family 25 (mitochondrial carrier; adenine nucleotide translocator), member 4	21,881	0.6	51.0	1,270	0.5	2,037	0.8
*ACTN3*	Actinin, alpha 3	21,097	0.6	51.6	2,042	0.7	1,264	0.5
*PGAM2*	Phosphoglycerate mutase 2 (muscle)	21,059	0.6	52.2	1,736	0.6	1,521	0.6
*RPS13*	Ribosomal protein S13	19,063	0.6	52.8	1,610	0.6	1,343	0.5

### Differential gene expression following training

Following correction for multiple testing, a total of 92 transcripts were significantly (FDR = 0.05) differentially expressed in the skeletal muscle transcriptome following a ten month period of training: nineteen transcripts showed increased expression (+0.72-fold to +29.3-fold), and 73 displayed decreased expression (-0.43-fold to -4.2-fold). Twenty of the differentially expressed transcripts lay within annotated genes, 54 transcripts were located < 5 kb up or downstream of annotated genes and for 18 transcripts no annotated genes were located within 5 kb. The transcripts located in the vicinity of equine genes may represent regulatory regions of the genes and more in-depth analysis and annotation of the recently sequenced equine genome may lead to a reassessment of the boundaries of many currently annotated genes [63]. The uncharacterised transcripts that were not in the region of any known equine gene may represent novel equine exercise related genes or non-protein coding regulatory mRNAs. The differentially expressed transcripts, including those located within 5 kb of a known gene, and the associated gene names are listed in Table 3 and Table 4.

**Table 3:** Genes significantly up-regulated post-training compared to pre-training levels

Tag	Gene symbol	Gene name	FC	Adj *P*
gctgctctgcagtctga	*ACADVL*	Acyl-Coenzyme A dehydrogenase, very long chain	0.72	0.037
gaataattgaagactgg	*ACTR3B*	Arp3 Actin-Related Protein 3 Homolog B	2.37	0.041
gcgtccttgaggtccgg	C14orf153*	Chromosome 14 open reading frame 153	1.14	0.049
ctgtttttctgtttttt	CUL3*	Cullin 3	1.43	0.004
tgataccaatattcagt	FBXO32*	F-box protein 32	1.17	0.009
cagaaagagcagggaag	GOT1*	Glutamic-oxaloacetic transaminase 1, soluble	1.46	0.035
tggatgtgtggctatgg	*GRHPR*	Glyoxylate Reductase/Hydroxypyruvate Reductase	1.45	0.043
ggacccatgaaggacca	IGFN1*	Immunoglobulin-like and fibronectin type III domain-containing protein 1	2.03	0.019
tggttctgtttgttttg	KIAA1303*	P150 target of rapamycin (TOR)-scaffold protein	2.70	0.049
gagtgcagcctttcacc	*MRPS21*	28 S ribosomal protein S21, mitochondrial	3.24	0.024
accagagagatgaatgt	*MRPS21*	28 S ribosomal protein S21, mitochondrial	1.76	0.032
tgttgaagcgatgcagt	PER2*	Period homolog 2	29.26	0.004
tgttggtaagtagatcg	PER3*	Period homolog 3 (Drosophila)	1.05	0.047
tggctgtatggggaggc	SLC25A29*	Solute carrier family 25 member 29	1.32	0.013
gccttctgcacccagaa	TNNT3*	Troponin T Type 3 (Skeletal, Fast)	1.55	0.002
ttaaatatacttggaag	ZAK*	Sterile alpha motif and leucine zipper containing kinase AZK	2.08	0.023

An asterisk (*) indicates that the tag lay within 5 kb of the gene. FC - fold change; Adj P - adjusted *P *value using the Benjamini and Hochberg method.

**Table 4:** Genes significantly down-regulated post-training compared to pre-training levels

Tag	Gene Symbol	Gene Name	FC	Adj *P*
acccgagagacagccga	*ACTN3*	actinin, alpha 3	-0.97	0.029
acacagttagttaattt	AHCYL2*	Putative adenosylhomocysteinase 3	-1.39	0.006
acacagttagttaattt	AHCYL2*	Putative adenosylhomocysteinase 3	-1.39	0.006
taattttatttttttta	ANKHD1*	Ankyrin repeat and KH domain containing 1	-2.30	0.043
ttttccctcacatcttc	APOOL*	Apolipoprotein O-like	-0.54	0.003
cttagtgtgtatatctc	*ATP2B1*	ATPase, Ca++ transporting, plasma membrane 1	-0.62	0.041
atcattattttaccttt	BCL6*	B-cell CLL/lymphoma 6	-3.09	0.011
acggttttccccagatc	C1orf51*	Chromosome 1 open reading frame 51	-1.61	0.035
cactggccaaaagattt	*C21orf7*	Chromosome 21 open reading frame 7	-3.08	0.035
ccactaccctcttactc	CALM3*	Calmodulin3	-1.46	0.009
acagacacttggctaaa	CALM3*	Calmodulin3	-0.83	0.043
aacagaatcaaggagct	*CCNDBP1*	Cyclin-D1-binding protein 1	-0.81	0.035
gaaaacagtagctaaag	*DAG1*	dystroglycan 1	-1.49	0.022
ctcaacagcaacatcaa	*EIF3F*	Eukaryotic translation initiation factor 3, subunit F	-0.52	0.002
tccagcctcaaagcatt	FBXL17*	F-Box And Leucine-Rich Repeat Protein 17	-0.52	0.002
aactgtagtgctttaaa	*GATM*	Glycine Amidinotransferase	-1.35	0.035
taggttttacctccatt	GATM*	Glycine Amidinotransferase	-1.35	0.001
ctggaacaggggcgaac	GLUL*	Glutamate-ammonia ligase	-3.94	0.007
cccatcatccccttcct	*GPSN2*	GLYCOPROTEIN, SYNAPTIC 2	-1.06	0.004
aagtcccaccccaatat	GSTM5*	Glutathione S-transferase M5	-1.1	0.004
tccacccataagcagat	HOXC9*	Homeobox protein Hox-C9	-1.04	0.038
ggactgtctttattttt	IGFBP5*	Insulin-like growth factor binding protein-5	-2.32	0.001
gtaaccctacacagtca	IGFBP5*	Insulin-like growth factor binding protein-5	-2.12	0.038
cccagaaagacatttgt	IRF2BP2*	Interferon regulatory factor 2 binding protein 2	-1.57	0.001
caaaaggctctcctaat	*KCMF1*	potassium channel modulatory factor 1	-0.81	0.048
tttccattcaacaaaaa	KPNA1*	Karyopherin alpha 1	-1.8	0.006
aattactctttcactgt	KPNA3*	Karyopherin alpha 3	-1.55	0.043
cttttcacacacaaaac	LRRFIP1*	Leucine Rich Repeat	-0.95	4.1 × 10^-5^
ttaagtgccattactac	*MAP3K4*	Mitogen-activated protein kinase kinase kinase 4	-0.85	0.048
ccccaccctactcccac	MLEC*	Malectin	-1.13	0.017
tatgacagaaaagcaac	MSTN*	Myostatin	-2.56	0.002
atgactgtataatgtga	MSTN*	Myostatin	-2.55	0.008
gttcctaaataaataat	MSTN*	Myostatin	-4.2	0.004
ctgctgagcggcctctc	*MYLK2*	Myosin Light Chain Kinase 2, Skeletal Muscle	-1.94	0.034
gctcattaaagaacaaa	MYO9A*	Myosin Ixa	-1.03	0.043
ctatcttttccttttct	NAT12*	N-acetyltransferase MAK3 homolog	-0.68	0.002
attgtttaaatatcact	NEDD4*	Neural precursor cell expressed, developmentally down-regulated 4	-0.97	0.012
aaatcccaccctcccct	NLN*	Neurolysin	-1.02	0.008
tccagctttctattctt	PALLD*	Palladin, cytoskeletal associated protein	-1.06	0.039
cttctttccccacctcc	*PCBP4*	Poly(rC) binding protein 4	-0.82	0.004
taattgcagtttactat	GNPNAT1*	Similar to glucosamine-phosphate N-acetyltransferase 1	-1.23	0.006
ctctagaacatttacct	PTDSS1*	Phosphatidylserine synthase 1	-0.62	0.003
agagtcaatataaaggt	PTPLA*	Protein Tyrosine Phosphatase-Like	-2.07	0.01
aaatctaaagttaaata	RAB33B*	RAB33B, member RAS oncogene family	-1.24	0.04
cagccctgggggcctac	*RYK*	Ryk Receptor-Like Tyrosine Kinase	-0.94	0.005
attcccatttctagtaa	SEPT7*	Transcribed locus	-0.72	0.018
tgaggacaaagctcagg	SLC10A2*	Solute carrier family 10	-4.11	0.001
ccttcaactcaacaaat	SNAP29*	Synaptosomal-associated protein, 29 kDa	-0.43	0.009
gtattgtaagatattaa	SNORA24*	Small nucleolar RNA SNORA24	-0.89	0.037
aagcaagaaataaattt	TET1*	Tet oncogene 1	-1.4	0.029
atgcagagcaccacaga	*TLE1*	transducin-like enhancer of split 1	-1.58	2.0 × 10^-5^
actgacatatgtaaaga	TOR3A*	torsin family 3, member A	-1.49	0.022
tgcctatcacctgccgg	TPD52*	Tumor protein D52	-0.68	0.005
tcctttcagtcttcaca	*TSPAN3*	Tetraspanin 3	-0.58	0.04
ctttgtgacttccaagt	ULK2*	Unc-51-like kinase 2	-0.72	0.018
aaaccatatttcttccc	VCL*	Vinculin	-1.13	0.005
cccaggcccgtccctgc	ZC3H3*	Zinc finger CCCH-type containing 3	-1.28	0.035
aagtctaacttccattt	ZNF704*	Zinc finger protein 704	-0.93	0.013
ttagtttcttttcttta	ZWINT*	ZW10 interactor	-1.21	1.1 × 10^-4^

An asterisk (*) indicates that the tag lay within 5 kb of the gene. FC - fold change; Adj P - adjusted *P *value using the Benjamini and Hochberg method.

Genes with higher post-training basal mRNA levels included those involved in the mitochondria, ubiquitination and circadian rhythm regulation, whereas genes with significantly reduced mRNA were mainly associated with cytoskeletal structure and the control of growth and development.

### Functional profiling of differentially expressed genes

Significantly up and down-regulated blocks of functionally related genes were identified by performing a gene enrichment test (FatiScan) for all expressed genes ranked according to differential expression following training. Among the up-regulated genes we identified 275 significantly (FDR < 0.05) overrepresented GO terms and 13 KEGG pathways. Among the down-regulated genes we identified 207 significantly (FDR < 0.05) overrepresented GO terms and five KEGG pathways. Subsets of the functional groups are shown in Table 5 and Table 6, and were chosen to include all significant (FDR<0.05) KEGG pathways and all highly significantly (FDR < 0.0001) overrepresented functional groups satisfying GO > level 6 and for which at least six genes were identified.

**Table 5:** Gene ontology categories with significantly increased expression post-training compared to pre-training levels

GO ID	GO Term	*P*
BP:0009060	aerobic respiration	1.89 × 10^-16^
BP:0046356	acetyl-CoA catabolic process	2.28 × 10^-16^
BP:0006099	tricarboxylic acid cycle	5.83 × 10^-16^
BP:0006100	tricarboxylic acid cycle intermediate metabolic process	5.92 × 10^-10^
BP:0019395	fatty acid oxidation	4.65 × 10^-08^
BP:0006119	oxidative phosphorylation	3.69 × 10^-06^
BP:0006956	complement activation	4.07 × 10^-06^
BP:0002455	humoral immune response mediated by circulating immunoglobulin	4.07 × 10^-06^
BP:0002253	activation of immune response	5.31 × 10^-06^
BP:0006635	fatty acid beta-oxidation	6.70 × 10^-06^
BP:0002541	activation of plasma proteins during acute inflammatory response	8.46 × 10^-06^
BP:0050778	positive regulation of immune response	8.46 × 10^-06^
BP:0006631	fatty acid metabolic process	7.12 × 10^-05^
CC:0005739	mitochondrion	1.04 × 10^-41^
CC:0043231	intracellular membrane-bound organelle	8.17 × 10^-21^
CC:0019866	organelle inner membrane	2.16 × 10^-19^
CC:0044429	mitochondrial part	1.04 × 10^-16^
CC:0044444	cytoplasmic part	6.14 × 10^-16^
CC:0031967	organelle envelope	1.07 × 10^-08^
CC:0031090	organelle membrane	2.25 × 10^-05^
CC:0000793	condensed chromosome	5.40 × 10^-05^
CC:0005840	ribosome	7.74 × 10^-05^

**KEGG ID**	**KEGG pathway**	

hsa04510	Complement and coagulation cascades	5.61 × 10^-06^
has00750	Vitamin B6 metabolism	1.21 × 10^-04^
hsa00790	Folate biosynthesis	1.35 × 10^-04^
hsa00350	Tyrosine metabolism	3.93 × 10^-04^
hsa00760	Nicotinate and nicotinamide metabolism	4.51 × 10^-04^
hsa00020	Citrate cycle (TCA cycle)	0.002
hsa00190	Oxidative phosphorylation	0.002
hsa00500	Starch and sucrose metabolism	0.002
hsa00280	Valine, leucine and isoleucine degradation	0.003
hsa00380	Tryptophan metabolism	0.003
has00720	Reductive carboxylate cycle (CO2 fixation)	0.007
hsa00860	Porphyrin and chlorophyll metabolism	0.019
hsa00252	Alanine and aspartate metabolism	0.032

**Table 6:** Gene ontology categories with significantly decreased expression post-training compared to pre-training levels

GO ID	GO Term	*P*
BP:0006817	phosphate transport	2.92 × 10^-20^
BP:0015698	inorganic anion transport	7.84 × 10^-18^
BP:0050679	positive regulation of epithelial cell proliferation	9.57 × 10^-05^
CC:0005856	cytoskeleton	4.00 × 10^-09^
CC:0044430	cytoskeletal part	5.59 × 10^-09^
CC:0016528	sarcoplasm	6.32 × 10^-07^
CC:0015629	actin cytoskeleton	2.11 × 10^-06^
CC:0016529	sarcoplasmic reticulum	2.93 × 10^-06^
CC:0005887	integral to plasma membrane	6.96 × 10^-06^

**KEGG ID**	**KEGG pathway**	***P***

hsa04530	Tight junction	5.30 × 10^-04^
hsa04630	Jak-STAT signaling pathway	0.008
hsa04720	Long-term potentiation	0.013
hsa01430	Cell communication	0.032
hsa04512	ECM-receptor interaction	0.046

The most significantly overrepresented cellular compartment GO groups among the genes with increased abundance post-training were mitochondrion (CC GO:0005739; *P *< 1.04 × 10^-41^) and related terms such as organelle inner membrane (CC GO:0019866) and mitochondrial part (CC GO:0044429). Aerobic respiration (BP GO:0009060), oxidative phosphorylation (BP GO:0006119) and the tricarboxylic acid cycle (GO BP:0006099) were among the overrepresented GO biological processes groups. The KEGG pathways included Citrate cycle (TCA cycle) (hsa00020) and Oxidative phosphorylation (hsa00190) and multiple metabolism pathways. These transcriptional data concur with biochemical and physiological studies that have demonstrated an increase in mitochondrial volume and aerobic capacity following endurance training [1,2]. Although there is evidence to indicate that an increase in oxidative capacity is part of the maturation process in horses [64] it has been demonstrated that exercise training, not growth, causes increases in whole muscle activity of the oxidative enzyme succinate dehydrogynase and changes in muscle fibre type composition in young Thoroughbred horses [14]. To our knowledge, this is the first time that these GO groups have been shown to have increased expression following exercise training.

This highlights the value of using a method such as FatiScan which incorporates all experimental data rather than limiting interpretation to those that rank among the highly differentially expressed. Only three mitochondrial genes were among those significantly differentially expressed: *MRPS21*, *SLC25A29 *and *ACADVL*. *MRPS21 *is a nuclear-encoded mitochondrial ribosomal gene required for protein synthesis in the mitochondria. Therefore, an increase in mitochondrial abundance would require an increase in mitochondrial protein synthesis. The *SLC25A29 *and *ACADVL *proteins are localized in the mitochondrial inner membrane and play a role in fat metabolism [65,66]. The fatty acid oxidation (BP GO:0019395), fatty acid beta-oxidation (BP GO:0006635) and fatty acid metabolic process (BP GO:0006631) GO ontologies were also overrepresented among genes that increased expression following training. This is in agreement with previous observations of a shift towards fatty acid metabolism in response to exercise training [3]. Furthermore, 12 of the 13 up-regulated KEGG pathways were associated with aerobic respiration and metabolism. Overall these results demonstrate that exercise training brings about a subtle but coordinated increase in the basal level of gene expression of a wide array of genes involved in energy production and metabolism.

Interestingly there was also an up-regulation of GO terms involved in the immune response such as the KEGG pathway complement and coagulation cascades, complement activation and positive regulation of immune response. The up-regulation of the complement and coagulation cascades may be a response to exercise induced hemolysis. It has been suggested that exercise induced decreases in blood pH and increases in blood temperature may increase the osmotic fragility of erythrocytes. Previous studies have shown that an immune response is elicited in response to a single bout of exercise and that this response is attenuated in trained subjects. Furthermore, it appears that moderate exercise can enhance the immune response [67], whereas over-training in humans is detrimental to health and can leave athletes more susceptible to infection [68]. Overtraining in horses has been associated with increased levels of the alpha-1-antitrypsin protein [69] which is involved in protection of cells from inflammatory enzymes released from neutrophils [70]. This protein was also found to be increased in humans following a marathon run but returned to basal levels within a few hours [71]. Despite numerous studies documenting the immune response to a single bout of exercise [72-74], little is known regarding the molecular mechanisms governing the adaptations to the immune response brought about by exercise training. It has been suggested that exercise-induced reactive oxygen species (ROS) may play a major role in the modulation of the immune response following exercise [75]. It is also likely that exercise-induced muscle damage contributes to the inflammatory response [76]. The exercise regime undertaken by the horses in this study incorporated both endurance and sprint work which would be expected to elicit both increased ROS and intramuscular microtears.

Another interesting observation was the increased expression of ribosomal genes as elevated rates of protein synthesis and degradation have been reported following resistance exercise with an overall increase in protein mass [4,77,78].

The down-regulated functional groups were mainly associated with structural genes and ion transport. It has been shown that the cellular response to mechanical stimuli, such as increased load, involves ECM signalling to the cytoskeleton at focal adhesion complexes via integrin receptors. Ion transport is central to muscular contraction. Calcium is the main regulatory and signalling molecule in muscle and ATP synthesis is dependent on phosphate transport. Although the down-regulation of these functional groups is counter-intuitive, the modulation of gene expression in these functional groups may reflect structural reorganization of myofibrils.

### Validation of a panel of genes by real time qRT-PCR

Eleven genes represented by tags that were differentially expressed between untrained and trained skeletal muscle were selected for real time qRT-PCR validation. Four tags (acyl-coenzyme A dehydrogenase, very long chain [*ACADVL*], actinin, alpha 3 [*ACTN3*], dystroglycan 1 [*DAG1*] and 28 S ribosomal protein S21, mitochondrial [*MRPS21*]) were located within a known gene and seven (calmodulin 3 [*CALM3*], insulin-like growth factor binding protein-5 [*IGFPB5*], myostatin [*MSTN*], period homolog 2 [*PER2*], period homolog 3 [*PER3*], solute carrier family 25 member 29 [*SLC25A29*] and troponin T type 3 [*TNNT3*]) were located within 5 kb of a known gene and were predicted to represent the gene. Primers were designed to span exons 1 and 2 or exons 2 and 3 of the gene of interest. This approach was taken to validate both the differential expression of genes and to assess the prediction that the differentially expressed tags that were identified within 5 kb of a known gene were indeed representative transcripts of that gene.

The mean expression of three of the four genes represented by intergenic tags reached significance (*P *< 0.05) and concurred with DGE data. *ACTN3 *showed the same direction of change as the DGE data and tended towards significance (*P *< 0.1). The mean expression of six of the seven genes predicted to be represented by adjacent tags agreed with the DGE data, the exception being *TNNT3*. The putative *TNNT3 *tag was matched to a region ~880 bp downstream of the *TNNT3 *gene and may represent a novel gene or mRNA. Alternatively the tag may span a splice site in an alternative gene and consequently may represent RNA transcribed from a different region in the genome. Real time qRT-PCR results are detailed in Table 7.

**Table 7:** Real time qRT-PCR results for genes used to validate DGE data

Tag	Gene Symbol	Gene Name	DGE FC	RT-PCR FC	P
gctgctctgcagtctga	*ACADVL*	Acyl-Coenzyme A dehydrogenase, very long chain	1.65	1.72	0.014
acccgagagacagccga	*ACTN3*	Actinin, alpha 3	-1.97	-1.41	NS
ccactaccctcttactc	*CALM3*	Calmodulin 3	-2.75	-1.81	0.028
gaaaacagtagctaaag	*DAG1*	Dystroglycan 1	-2.81	-1.27	0.021
ggactgtctttattttt	*IGFBP5*	Insulin-like growth factor binding protein-5	-4.35	-3.18	0.023
gagtgcagcctttcacc	*MRPS21*	28 S ribosomal protein S21, mitochondrial	9.47	6.03	0.013
tatgacagaaaagcaac	*MSTN*	Myostatin	-4.35	-4.97	0.004
tgttgaagcgatgcagt	*PER2*	Period homolog 2	+inf	1.88	0.003
tgttggtaagtagatcg	*PER3*	Period homolog 3 (Drosophila)	2.07	1.74	0.001
tggctgtatggggaggc	*SLC25A29*	Solute carrier family 25 member 29	2.50	1.22	0.035
gatgaagctgggatgca	*TNNT3*	Troponin T Type 3	2.93	0.94	NS

The figure for the DGE fold change could not be calculated due to the fact that the PER2 transcript was undetectable pre-training (the transcript had zero counts in all the pre-training samples).

The *PER2 *and *PER3 *genes, key molecular clock components within the mammalian circadian timing system [79], had mean post-training increases in expression of +1.88-fold and +1.74 fold respectively. The induction of these genes may represent an entrainment of the muscle transcriptional clock by a regular exercise regime. While primarily regulated by photoperiodic signals to the master pacemaker within the suprachiasmatic nucleus, peripheral circadian clocks, which are known to exist in almost all peripheral tissues examined to date [80], can also be entrained by alternative timing cues including exercise [81] and feeding [82]. The role of peripheral clocks is to align specific tissue function to the correct time of day via differential regulation of subsets of clock-controlled genes.

As exercise is a known synchroniser of circadian rhythms in mice [83], humans [81] and horses [84], and *PER2 *has previously been shown to oscillate in equine tissues [85], the increased expression of PER genes post-training in the current study is thought to represent a strengthening of the endogenous circadian clock in equine muscle. Furthermore, human studies have shown time of day variations in exercise performance at the physiological level [86-88], and it has been suggested that circadian rhythms may play an important role in sports performance [89]. Combined with our results, this is strong incentive for further investigation of the influence of training times on daily muscle function in the horse, such that optimal athletic performance may be achieved.

The proteins encoded by *ACADVL *(+1.72-fold, *P *= 0.014), *MRPS21 *(+6.03-fold, *P *= 0.013) and *SLC25A29 *(+1.22-fold, *P *= 0.350) function in the mitochondria to increase protein synthesis and fat metabolism. The increase in expression of the gene encoding the mitochondrial ribosomal protein MRPS21 likely reflects an increase in mitochondrial protein synthesis and an overall increase in mitochondrial volume. Numerous studies have demonstrated an increase in mitochondrial volume concurrent with an increase in VO_2max _following endurance training [90-93]. The proteins encoded by *ACADVL *and *SLC25A29 *are involved in fat metabolism and are located in the mitochondrial inner membrane.

*ACTN3*, *CALM3 *and *DAG1 *were decreased in expression by -1.41-fold (*P *= 0.090), -1.81-fold (P = 0.028) and -1.27-fold (*P *= 0.021) respectively. The ACTN3 protein is localized to the skeletal muscle z-discs and DAG1 forms part of the dystroglycan complex. A null mutation in the *ACTN3 *gene has been associated with sprint performance in human athletes [94] and *DAG1 *has been proposed as a candidate gene in some muscular myopathies [95,96]. CALM3 is an isoform of calmodulin, a calcium-modulated protein which regulate numerous protein targets. The binding of calcium to calmodulin induces a conformational change which affects its ability to bind target proteins. In this manner calmodulin may be used by other proteins as a calcium sensor and signal transducer. CALM3 may be involved in muscle fibre type transformation in response to muscle excitation [97,98]. *CALM3 *gene expression was also decreased in equine muscle four hours post exhaustive treadmill exercise [47].

*IGFBP5 *and *MSTN *encode growth factors with large observed decreases in expression post training (-3.18-fold, *P *= 0.023 and -4.97-fold *P *= 0.004 respectively). IGFBP5 is one of family of modulators of insulin like growth factors (IGFs) which interact with IGFs resulting in an increase in half life and alteration of the interaction with receptors. IGF-1 promotes muscle hypertrophy and protein levels are increased in humans following administration of human growth hormone as an illegal doping agent [99,100]. The exact mode of action of IGFBP5 is poorly understood however it has been shown to associate with the extra cellular matrix and is a regulator of a wide range of physiological processes including cell proliferation and muscle cell differentiation [101-103].

Myostatin encoded by the *MSTN *gene is a negative regulator of muscle growth and an inhibitor of satellite cell proliferation[104]. The expression of *MSTN *was found to be decreased in humans following resistance training [105,106]. Null mutations in this gene have been found to cause a double muscling phenotype in cattle, dogs, and humans [107-111]. Structural variation in the *MSTN *gene has also been associated with athletic performance in dogs [110] and horses [112]. The differential expression of this gene is of particular significance as an intronic SNP in equine *MSTN *has been found to be a strong predictor of optimal racing distance in Thoroughbred racehorses [112].

## Conclusion

Deep sequencing of the equine skeletal muscle transcriptome has revealed novel transcripts and functional groups associated with this tissue. Furthermore, following exercise training we have observed an increase in the occurrence of genes involved in metabolism and oxidative phosphorylation, and a decrease in the expression of structural genes. Overrepresented functional groups of genes post-training were associated with both endurance and resistance exercise. This study documents the transcriptome-wide reprogramming of skeletal muscle in Thoroughbred racehorses that brings about the well documented phenotypic adaptations to exercise.

## Methods

### Subjects

All animal procedures were approved by the University College Dublin, Animal Research Ethics Committee, a licence was granted from the Department of Health and Children (Ireland) and owners' consent was obtained for all horses.

Seven two-year-old untrained Thoroughbred horses (*n *= 5 females, *n *= 2 entire males), raised on the same farm for the previous 2 - 3 months and destined for Flat racing with the same trainer comprised the study cohort. The horses had a mean height of 154.9 cm (± 2.8) and a mean pre-training weight of 437.4 kg (± 18.0). All horses undertook a regular exercise regime with the same trainer for 10 months (trained). This consisted of light canter (1,500 m) once a day six times a week on an all-weather gallop and higher intensity exercise ("work") no more than once a week which consisted of warm-up (walk and trot) followed by gallop with velocities reaching maximal intensity for 800-1,000 m.

### Muscle biopsy sampling

Percutaneous needle muscle biopsies [113] were obtained from the dorsal compartment of the *gluteus medius *muscle according to Dingboom and colleagues [114] using a 6 mm diameter, modified Bergstrom biopsy needle (Jørgen KRUUSE, Veterinary Supplies). Biopsies were taken approximately 15 cm caudodorsal (one-third of the distance) to the *tuber coxae *on an imaginary line drawn from the *tuber coxae *to the head of the tail. The biopsies were obtained at a depth of 80 mm. Each biopsy site was shaved, scrubbed with an antiseptic and desensitized by a local anaesthetic. The biopsy samples were washed with sterile PBS (BD Biosciences, San Jose, CA) and preserved in RNA*later *(Ambion, UK) for 24 hours at 4°C and then stored at -20°C. Muscle biopsy samples were collected at rest at two time points: T_0_-untrained and T_2_-trained.

### RNA isolation and purification

Approximately 100 mg of each muscle biopsy sample was removed from RNA*later *and homogenized in 1 ml TRIzol using a TissueLyser (Qiagen Ltd, Crawley, UK) and extracted according to the manufacturer's instructions. Each sample was purified using the RNeasy^® ^Mini kit (Qiagen Ltd, Crawley, UK) and DNase treated with RNase free DNase (Qiagen Ltd, Crawley, UK). RNA was quantified using a NanoDrop^® ^ND1000 spectrophotometer V 3.5.2 (NanoDrop Technologies, Wilmington, DE) and RNA quality was subsequently assessed using the 18S/28 S ratio and RNA integrity number (RIN) on an Agilent Bioanalyser with the RNA 6000 Nano LabChip kit (Agilent Technologies Ireland Ltd, Dublin, Ireland) according to the manufacturers' instructions.

### Library preparation for Illumina sequencing

The Illumina cDNA library was prepared according to the manufacturer's instructions. All reagents were supplied by Illumina apart from SuperScript II Reverse Transcriptase (part # 18064-014) with 100 mM DTT. Briefly, 1.5 μg mRNA was isolated from total RNA by binding the mRNA to a magnetic oligo(dT) bead. Double stranded cDNA was synthesized and cleaved at each *Nla*III site. The site of *Nla*III cleavage was ligated with an Illumina-supplied adaptor using T4 DNA ligase. The bead bound double stranded cDNA was the cut by the restriction enzyme, *Mme*I. This resulted in a 17 bp tag which was no longer attached to the oligo(dT) bead. The cDNA construct was then precipitated and the site of *Mme*I cleavage was ligated with an Illumina-supplied adaptor using T4 DNA ligase. The adaptor ligated cDNA was PCR amplified with two adapter primers (Illumina). The PCR product of 85 bp was purified by gel extraction in preparation for loading on the Illumina Cluster Station. The quality and quantity of the purified constructs were assessed using an Agilent DNA series 7500 series II assay (Agilent Technologies Ireland Ltd, Dublin, Ireland) and Qubit fluorometer according to manufacturer's instructions. Cluster generation and sequencing analysis were carried out using Illumina's Solexa Sequencer according to the manufacturer's instructions.

### Analysis

The DGE samples were processed through the standard software pipeline provided by Illumina for the Genome Analyzer. The sequence reads were base called using the Bustard base caller (part of the Illumina software). The tag annotation pipeline consisted of two parts: mapping to known Ensembl [115] cDNAs and mapping to the genome. The known cDNAs from version 49 of Ensembl for the EquCab2 assembly of the equine genome were downloaded in FASTA format using the Ensembl biomart tool. The FASTA files for the individual equine chromosomes were downloaded from the UCSC genome browser website [116]. A pipeline consisting of perl, C++ and linux shell scripts was used to conduct an *in-silico *digestion of both the transcriptome and genome and to generate tag location records which were loaded into a MySQL database. The tag records were then annotated according to their type (genomic or cDNA, canonical, noncanonical, repeat etc.). A matrix of tag counts for each sample was generated. The edgeR Bioconductor package [117] was used to determine differential expression of tags in each group.

### Functional clustering according to gene ontology annotations

The equine Ensembl gene IDs were cross-matched to human Ensembl gene IDs. Using the Ensembl IDs of human homologues of equine genes it was possible to use the Database for Annotation, Visualization and Integrated Discovery (DAVID) [118,119] for functional clustering and overrepresentation analyses. The Expression Analysis Systematic Explorer (EASE) tool [120] within DAVID was used to investigate the representation of functional groups in equine skeletal muscle relative to the whole genome. The FatiScan [121,122] gene enrichment test was used to analyse the transcriptional profile post-training. FatiScan is part of the Babelomics Suite of web tools and tests for the asymmetrical distribution of biological labels in an ordered list of genes through application of a Fisher's exact test. Genes were ranked by differential expression and FatiScan was used to detect functional blocks (GO and KEGG pathways) that were significantly up-regulated and down-regulated post-training. Results from both EASE and FatiScan were corrected for multiple testing using the Benjamini and Hochberg method [123].

### Real time quantitative RT-PCR

Selected cDNA samples were quantified by real time quantitative RT-PCR (qRT-PCR). 1 μg of total RNA from each sample was reverse transcribed into cDNA with oligo-dT primers using a SuperScript™ III first strand synthesis SuperMix kit according to the manufacturer's instructions (Invitrogen Ltd, Paisley, UK). The converted cDNA was diluted to 2.5 ng/μl working stocks and stored at -20°C for subsequent analyses. Oligonucleotide primers for real time qRT-PCR were designed using Primer3 version 3.0 http://www.primer3.sourceforge.net and commercially synthesized (MWG Biotech, Germany). Primer details are shown in Table 8. Each reaction was carried out in a total volume of 20 μl with 5 μl of cDNA (1 ng/μl), 10 μl SYBR^® ^Green PCR Master Mix (Applied Biosystems, Cambridgeshire, UK) and 5 μl primer/H_2_O. Real time qRT-PCR was performed using a 7500 Fast Real-Time PCR machine (Applied Biosystems, Cambridgeshire, UK). All reactions were performed in duplicate. Hypoxanthine phosphoribosyltransferase 1 (*HPRT*) was selected as a stable reference gene based on a study of equine reference genes for real time qRT-PCR [124] and on the DGE results. Expression values were calculated using a standard curve which was plotted based on the expression of *HPRT *in serial dilutions of equine skeletal muscle RNA (1:1, 1:2, 1:4, 1:8, 1:16, 1:32, and 1:64). The standard curve method was used to normalise the gene expression data. The paired Student's t-test was used to identify significant differences in mRNA abundance between time-points.

**Table 8:** Real time qRT-PCR primers for genes used to validate DGE data

Gene symbol	Forward Primer	Reverse Primer
*ACADVL*	ctgcccagcgatcctatg	ttccactggtcgaagtctca
*ACTN3*	cggcgagtatatggaacagg	gtgagttgcaccaggcagt
*CALM*	agcacttggtggactccttg	aaatgcctgactgtgctcaa
*DAG1*	ccaggaggagtgagcacct	ctcaccctctgcacacctg
*IGFBP5*	ggaggagccgagaacactg	gcgaagcctccatgtgtc
*MRPS21*	ggagatctgctgtttgctca	tctctcaaagcgacccatct
*MSTN*	tgacagcagtgatggctctt	ttgggttttccttccacttg
*PER2*	agcctgatgatggcgaagtctgaa	agttctttgtgcgtgtctgccttg
*PER3*	aactatgcccttcgctgtgt	gtacccggtcacatctgctt
*SLC25A29*	ggacacccgtttgacactg	ctgatgatggattggaagca
*TNNT3*	cggagggggagaaagtagac	caaagtggctgtcgatgaga

## Authors' contributions

BMcG & EH designed the experiment. BMcG, EH, LK and RF participated in the collection of samples. BMcG performed the RNA extractions. BMcG and JB generated the cDNA libraries. BMcG performed the real time qRT-PCR experiments. PM was responsible for the bioinformatics pipeline. AL, BL and AE were responsible for the sequencing project. BMcG performed functional analysis of the data. PM, BM and DMacH assisted with manuscript preparation. BMcG and EH wrote the paper. EH coordinated and supervised the project. All authors read and approved the final manuscript.
